# Confirmation of canine acanthomatous ameloblastoma using RAS Q61R immunohistochemical staining of formalin-fixed paraffin-embedded tissues

**DOI:** 10.3389/fvets.2023.1281022

**Published:** 2023-10-13

**Authors:** Santiago Peralta, Magdalena M. Marcinczyk, William P. Katt, Gerald E. Duhamel

**Affiliations:** ^1^Department of Clinical Sciences, College of Veterinary Medicine, Cornell University, Ithaca, NY, United States; ^2^Department of Biomedical Sciences and New York Animal Health Diagnostic Center, College of Veterinary Medicine, Cornell University, Ithaca, NY, United States; ^3^Department of Molecular Medicine, College of Veterinary Medicine, Cornell University, Ithaca, NY, United States

**Keywords:** canine acanthomatous ameloblastoma, oral tumor, immunohistochemistry, HRAS, somatic mutation, SP174

## Abstract

Differentiating canine acanthomatous ameloblastoma (CAA) from oral squamous cell carcinoma (OSCC) based on routine histopathology can be challenging. We have previously shown that more than 95% of CAAs harbor an *HRAS* p.Q61R somatic mutation, while OSCCs carry either wild-type alleles or other MAPK pathway activating mutations (e.g., *HRAS* p.Q61L, *BRAF* p.V595E). Given that *HRAS* p.Q61R mutations are highly prevalent in CAA, we hypothesized that a RAS Q61R-specific rabbit monoclonal antibody may be a useful tool for confirmation of CAA by immunohistochemical (IHC) staining. In the present study, we assessed IHC staining of archived formalin-fixed and paraffin-embedded biopsy samples with a diagnosis of CAA (*n* = 23), using a RAS Q61R-specific rabbit monoclonal antibody (SP174) and an automated IHC stainer. Negative control samples consisted of *HRAS* p.Q61R mutation-negative OSCC tumors with either a known *HRAS* p.Q61L mutation (*n* = 1), *BRAF* p.V595E mutation (*n* = 4), or wild-type corresponding alleles (*n* = 3). We found that all 23 CAAs showed diffuse and strong membranous RAS Q61R immunoreactivity (100% sensitivity), while none of the 8 OSCCs showed immunoreactivity (100% specificity). The data supports the use of RAS Q61R-specific rabbit monoclonal antibody for diagnostic IHC confirmation of CAA and ruling out OSCC in dogs.

## 1. Introduction

Canine acanthomatous ameloblastoma (CAA) is a common neoplasm that originates from odontogenic epithelium (1–4). It has been reported in multiple breeds of dogs of a wide age range (mean ~ 9 years) without an apparent sex predilection (5, 6). The tumor is characterized by a discrete space-occupying mass arising from a dentated area, with a variable degree of invasion of underlying anatomical structures, including the jawbone (7, 8). The current treatment of choice for CAA is wide-margin excision (i.e., mandibulectomy or maxillectomy), which typically offers long-term remission but is technically complex and frequently results in occlusal dysfunction (9–12). Other treatment modalities have been described, including marginal excision, radiation therapy and intralesional bleomycin injections, but long-term remission rates are either low or variable, and side effects can be considerable (13–15).

Definitive diagnosis of CAA is based on routine histopathological examination (3, 16). However, due to overlapping clinical, radiological, and histological features between CAA and oral squamous cell carcinoma (OSCC), up to ~30% of cases may be misdiagnosed (6, 17). This has important clinical implications given fundamental differences in biological behavior including significantly more rapid growth as well as higher metastatic potential of OSCC relative to CAA (8, 18). Although some of the molecular underpinnings driving OSCC and CAA tumorigenesis appear to be similar (e.g., aberrant MAPK pathway signaling) (19), we have shown in previous studies that their mutational landscape is distinct (20). Importantly, we have found that more than 95% of CAAs harbor an *HRAS* p.Q61R somatic mutation, while OSCCs typically harbor corresponding wild-type (WT) alleles or other MAPK pathway activating mutations (e.g., *BRAF* p.V595E, *HRAS* p.Q61L) (20, 21). Arguably, identifying the mutational profile of tumor tissues is critical to distinguishing between these different epithelial cell tumors. However, genotyping experiments usually require molecular biology and/or sequencing capacity that are not readily available in standard diagnostic settings. Moreover, accurate diagnosis of CAA and differentiation from OSCC is critical to making informed clinical decisions and adequate prognostication early in the course of the disease.

Immunohistochemistry (IHC) is an alternative technique that does not require a highly specialized infrastructure and can be used to produce a signal that corresponds to a mutated protein, providing a means to infer point mutations (22, 23). For this, monoclonal antibodies offering high sensitivity and specificity are required. One commonly used antibody, which we hypothesized might suit this purpose, is designated SP174. This recombinant rabbit monoclonal antibody was originally designed to detect human RAS Q61R, and is commercially-available (24). Of note, SP174 cross-reacts with different isoforms of RAS, making it useful for screening for p.Q61R mutations in *NRAS*, *KRAS*, and *HRAS* (22–26). Given that canine and human RAS isoforms share 98–100% sequence homology and based on the high frequency of *HRAS* p.Q61R somatic mutations in CAA (20, 21, 27), we hypothesized that SP174 could detect RAS Q61R in CAA and rule out OSCC. Therefore, the aim of this study was to determine the sensitivity and specificity of IHC using SP174 to detect RAS Q61R in CAA tumor tissue and a lack of immunoreactivity with OSCC, including tumors that harbor a closely related *HRAS* p.Q61L mutation.

## 2. Methods

Representative formalin-fixed and paraffin-embedded (FFPE) tissue sections from archived CAA and OSCC cases were assessed by IHC staining for the presence of RAS Q61R immunoreactivity using standard protocols and an automated IHC stainer (Bond-Max automated IHC staining system; Leica). All samples were submitted as biopsy specimens, obtained between 2013 and 2021, that did not undergo decalcification to preserve critical antigenic epitopes. The diagnosis of CAA was made based on initial hematoxylin and eosin assessment by a board-certified veterinary pathologist. The presence of an *HRAS* p.Q61R mutation was confirmed in the majority of CAAs using molecular assays, as previously described (8, 20, 21, 28). Briefly, 4-μm tissue sections mounted on charged slides were deparaffinized (AR9222, Bond dewax solution; Leica), and after heat epitope retrieval (AR9640, Bond epitope retrieval solution 2; Leica) for 30–40 min, the slides were incubated with RAS Q61R-specific rabbit monoclonal antibody (SP174; ab227658; Abcam, Cambridge, MA, United States) raised against Ras mutated Q61R synthetic peptide (25) diluted 1:80 or 1:100 for 60 min followed by polymeric horseradish peroxidase (DS9800; Bond polymer refine detection; Leica) linker antibody conjugate detection system for 30 min, and hematoxylin counterstain (DS9390; Leica) for 5 min. Negative controls consisted of confirmed *HRAS* p.Q61R-negative OSCC FFPE tissues (20, 21). Positive immunoreactivity was identified as brown staining with diaminobenzidine (DAB). The IHC evaluation was performed independently by two pathologists (M.M.M. and G.E.D), while blinded to the results of previous molecular analyses and medical records.

The use of archived diagnostic material, and/or review and data collection from medical records of client-owned animals for the purposes of this study was approved by Cornell University’s Veterinary Clinical Studies Committee and was considered exempt from review by Cornell University’s Institutional Animal Care and Use Committee.

## 3. Results

**Figure 1:**
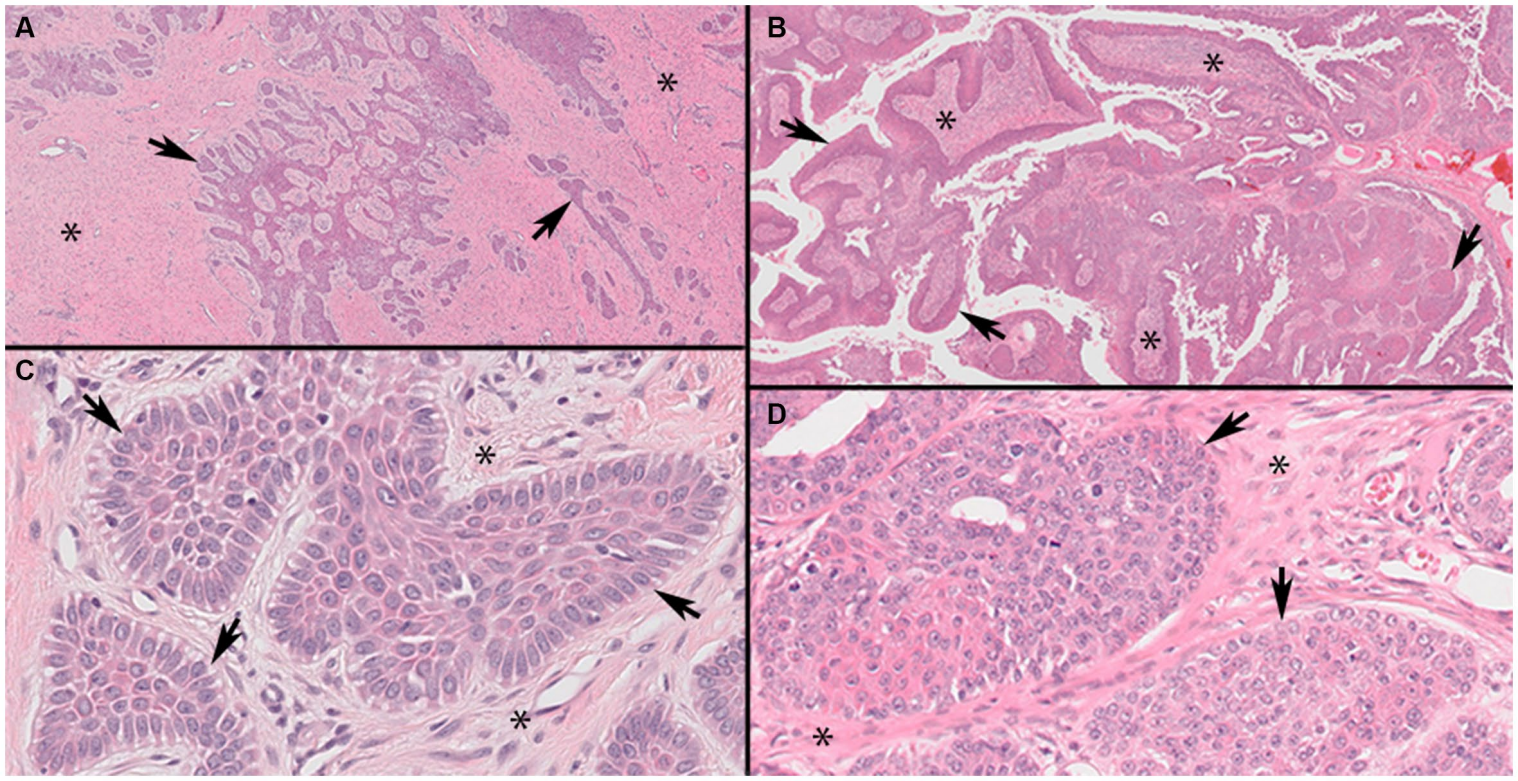
Representative photomicrographs of canine acanthomatous ameloblastoma (CAA) case 9 and oral squamous cell carcinoma, papillary subtype (pOSCC), case 27 (Panels **A,C**, ×4; **B,D**, ×40 original magnifications). Low magnification of CAA (Panel **A**) showing characteristic anastomosing cords and trabeculae of odontogenic epithelium (arrows) within a background of stellate mesenchyme (asterisks). Higher magnification of a CAA (Panel **C**) showing neoplastic islands with characteristic outer layer of epithelial cells oriented with their long axes perpendicular to the basement membrane (palisading) and anti-basilar nuclear polarization (arrows). Low magnification of a pOSCC (Panel **B**) showing characteristic exophytic papillary pattern consisting of broad trabeculae of neoplastic stratified squamous epithelium (arrows) supported by fibrovascular connective tissue stroma (asterisks). Higher magnification of a pOSCC (Panel **D**) showing irregularly arranged epithelial cells that lack anti-basilar nuclear polarization and indistinct epithelial to mesenchymal junction at the periphery of individual neoplastic epithelial lobules (arrows).

A total of 31 cases were enrolled representing 23 CAA and 8 OSCC tumors (Table 1; Figure 1). Of the 8 OSCC tumors, 5 corresponded to the papillary subtype (29). The average age was 8.85 ± 3.26 and 8.25 ± 3.11 for CAA and OSCC, respectively (*p* = 0.74, Mann–Whitney test). Sex distribution was 52% males and 48% with no significant differences based on tumor type (*p* = 0.92, Pearson’s chi-squared test). Seventeen dog breeds were represented with Labrador retriever and mixed breed dogs being the two most common, accounting for 22.6 and 12.9%, respectively. Of the 31 cases, 17 CAA and all 8 OSCC had been previously genotyped using RNA-seq, PCR and Sanger sequencing and/or restriction fragment length polymorphism experiments (20, 21). An *HRAS* p.Q61R somatic mutation was confirmed in all 17 genotyped CAA tumors. Only wild-type *HRAS* p.Q61 alleles were detected in 7 of the 8 OSCC tumors, and an *HRAS* p.Q61L mutation was detected in the remaining OSCC tumor. Four of the 8 OSCC tumors harbored a *BRAF* p.V595E mutation.

**Table 1:** Summary case information.

Case no.	Age (years)	Sex	Breed	Tumor location	Diagnosis	*HRAS* and *BRAF* status	SP174 immunostaining
1	7	MC	Great Dane	Rostral maxilla	CAA	*HRAS* p.Q61R	Positive
2	10	MC	Golden retriever	Caudal maxilla	CAA	*HRAS* p.Q61R	Positive
3	9	MC	Labrador retriever	Caudal mandible	CAA	*HRAS* p.Q61R	Positive
4	10	FS	Airedale terrier	Caudal mandible	CAA	*HRAS* p.Q61R	Positive
5	UNK	MC	Mixed	Rostral mandible	CAA	*HRAS* p.Q61R	Positive
6	11	FS	Labrador retriever	Caudal mandible	CAA	*HRAS* p.Q61R	Positive
7	12	MC	Mixed	Rostral mandible	CAA	*HRAS* p.Q61R	Positive
8	12	MC	Samoyed	Rostral maxilla	CAA	*HRAS* p.Q61R	Positive
9	12	FS	Labrador retriever	Rostral mandible	CAA	*HRAS* p.Q61R	Positive
10	9	FS	Staffordshire bull terrier	Caudal mandible	CAA	*HRAS* p.Q61R	Positive
11	4	FS	Poodle mix	Caudal maxilla	CAA	*HRAS* p.Q61R	Positive
12	11	MC	Shiba Inu	Rostral maxilla	CAA	*HRAS* p.Q61R	Positive
13	9	MC	Labrador retriever	Rostral mandible	CAA	*HRAS* p.Q61R	Positive
14	3	MC	Boxer	Rostral maxilla	CAA	*HRAS* p.Q61R	Positive
15	9	FS	Labrador retriever	Rostral mandible	CAA	*HRAS* p.Q61R	Positive
16	7	MC	Australian shepherd	Rostral maxilla	CAA	*HRAS* p.Q61R	Positive
17	7	FS	Bloodhound	Caudal maxilla	CAA	*HRAS* p.Q61R	Positive
18	0.6	M	Labrador retriever	Rostral mandible	CAA	ND	Positive
19	10	FS	Shetland sheepdog	Rostral maxilla	CAA	ND	Positive
20	15	MC	Beagle mix	Caudal mandible	CAA	ND	Positive
21	10	FS	Australian shepherd	Rostral maxilla	CAA	ND	Positive
22	10	FS	English cocker spaniel	Rostral mandible	CAA	ND	Positive
23	7	FS	Yorkshire terrier	Caudal maxilla	CAA	ND	Positive
24	5	MC	Mixed	Rostral mandible	OSCC	WT	Negative
25	10	FS	Yorkshire terrier	Rostral mandible	OSCC	WT	Negative
26	12	MC	Mixed	Rostral mandible	OSCC	WT	Negative
27	11	MC	Boxer	Rostral mandible	pOSCC	*HRAS* p.Q61L	Negative
28	5	MC	Great Dane	Rostral mandible	pOSCC	*BRAF* p.V595E	Negative
29	4	FS	Labradoodle	Caudal maxilla	pOSCC	*BRAF* p.V595E	Negative
30	9	FS	Labrador retriever	Rostral mandible	pOSCC	*BRAF* p.V595E	Negative
31	10	FS	Cocker spaniel	Rostral maxilla	pOSCC	*BRAF* p.V595E	Negative

MC, male castrated; FS, female spayed; M, male; CAA, canine acanthomatous ameloblastoma; OSCC, oral squamous cell carcinoma; pOSCC, papillary oral squamous cell carcinoma; ND, not determined; WT, wild-type.

**Figure 2:**
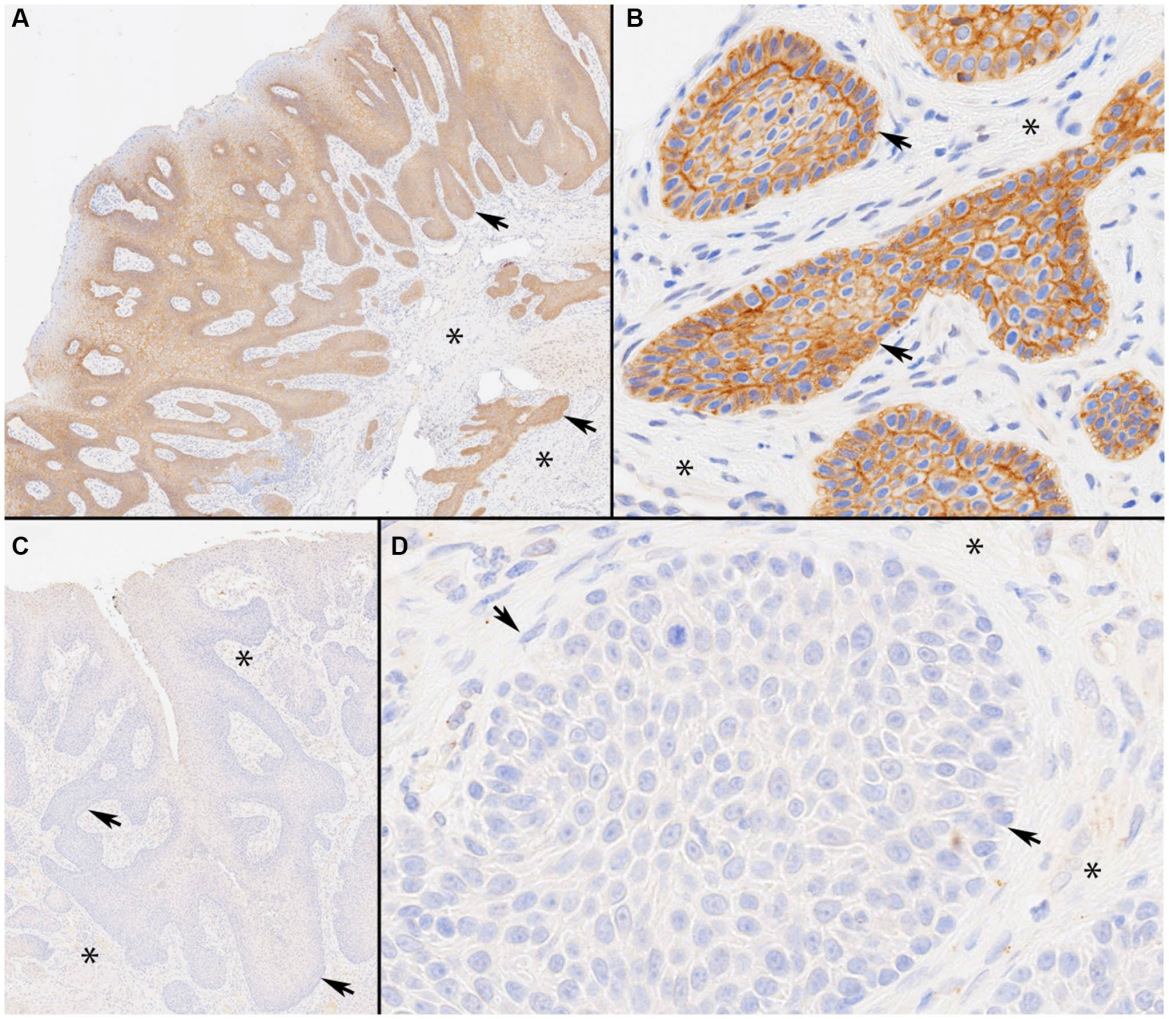
Immunolabeling of canine acanthomatous ameloblastoma (CAA) with RAS Q61R-specific rabbit monoclonal antibody (SP174). Photomicrographs of representative CAA case 9 with a confirmed *HRAS* p.Q61R mutation. Note strong membranous RAS immunoreactivity throughout CAA neoplastic trabeculae and islands (Panels **A**, ×4 and **B**, ×40 original magnifications). The staining highlights anti-basilar nuclear polarization in CAA (Panel **B**). By contrast, a representative oral squamous cell carcinoma (OSCC), papillary subtype, case 27 with a confirmed *HRAS* p.Q61L mutation, shows a lack of RAS immunoreactivity throughout (Panels **C**, ×4 and **D**, ×40 original magnifications). In each panel, asterisks represent stroma and arrows indicate neoplastic epithelial cells arranged in anastomosing trabeculae and lobules.

**Figure 3:**
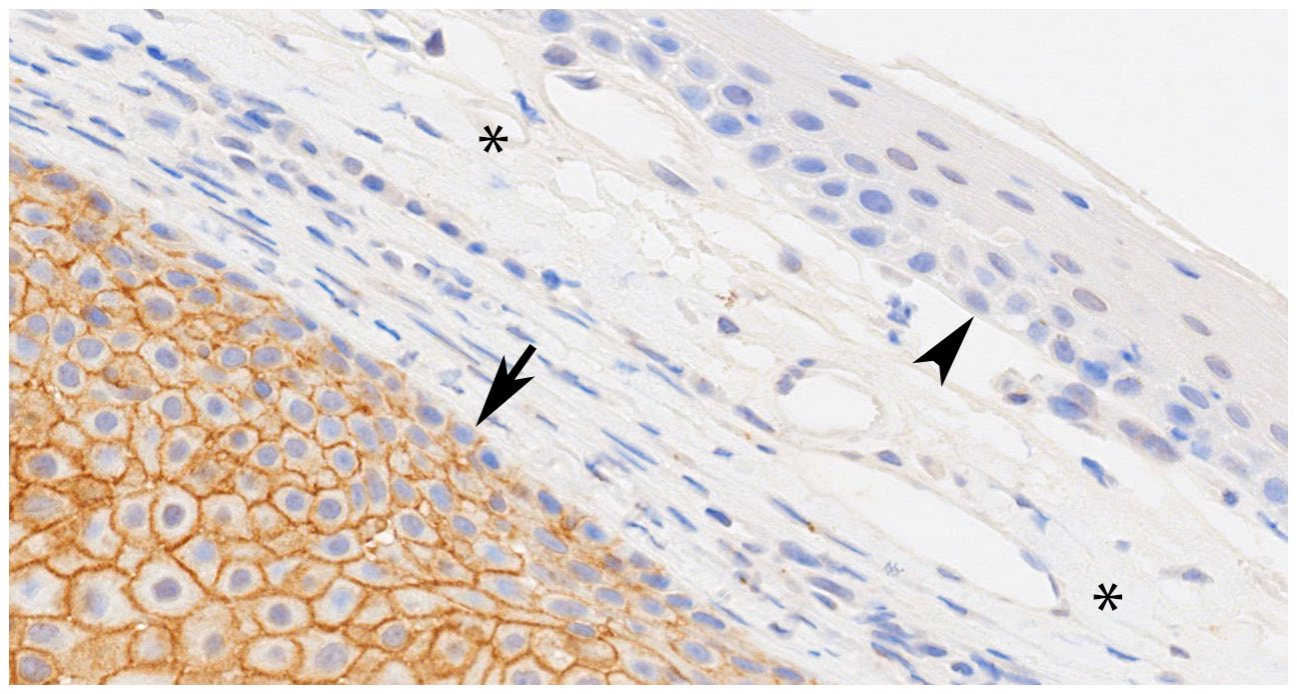
Representative photomicrograph of canine acanthomatous ameloblastoma (CAA), case 11 with confirmed *HRAS* p.Q61R mutation showing normal gingival epithelium (arrowhead) and connective tissue stroma (asterisks) without RAS immunoreactivity (internal negative control), while an underlying island of CAA neoplastic epithelial cells (arrow) shows strong membranous RAS immunoreactivity throughout (×40 original magnification).

All 23 CAAs showed diffuse and strong membranous RAS Q61R immunoreactivity of all neoplastic epithelial cells (100% sensitivity, Figure 2) consistent with the known membranous localization of RAS (https://www.proteinatlas.org/ENSG00000213281-NRAS) (30), while none of the 8 OSCCs showed immunoreactivity (100% specificity). Critical IHC parameters for optimal immunoreactivity included: (i) heat epitope retrieval for 30–40 min; less than 30 min showed positive but faint staining, (ii) 1:80 or 1:100 primary antibody dilution for 60 min, and (iii) Bound Polymer Refine Detection for 30 min; less than 30 min showed positive but faint staining. Repeated RAS Q61R IHC staining in three separate runs with serial sections taken from a subset of four CAA tissue samples yielded similar results (100% reproducibility). Normal gingival epithelium in a subset of CAA tissue samples (*n* = 9) showed no immunoreactivity, confirming RAS Q61R mutation is a reliable feature of CAA (Figure 3). The presence of strong immunoreactivity in a tissue sample taken from a 7-month-old dog with CAA indicates that like adult CAA, *HRAS* p.Q61R mutations likely underlie CAA in juvenile dogs. Unfortunately, a tissue sample from this juvenile dog was not available for genotyping.

## 4. Discussion

In this study, we assessed the utility of RAS Q61R-specific SP174 antibody for confirmation of CAA and distinction from OSCC. Given fundamental biological differences (6, 8, 18), accurate distinction between CAA and OSCC is essential for proper clinical decision making and prognostication. Results showed that SP174 cross-reacts with RAS Q61R in CAA FFPE tissues with 100% sensitivity and 100% specificity, allowing accurate distinction of these relatively common tumor types.

Other IHC strategies have been previously proposed to differentiate between CAA and OSCC (31, 32). For example, distinction based on cytokeratin and calretinin protein expression patterns has been suggested (32), but assessment relies purely on semi-quantitative scores and the diagnostic accuracy of these approaches has yet to be independently validated. Moreover, unbiased genomic profiling of CAA and OSCC shows variable expression patterns of the genes encoding these proteins without an obvious association with tumor type (19), suggesting that the patterns of cytokeratin and calretinin immunoreactivity in tumor tissues may not allow reliable tumor type distinction.

Another IHC-based strategy consists of determining the Ki67 labeling index of neoplastic epithelial cells (8, 33, 34). Ki67 is a nuclear protein expressed by cells that are actively engaged in the cell cycle (33). We have previously shown that the Ki67 labelling index of neoplastic cells is significantly lower in CAA compared to OSCC (8), which is a useful diagnostic complement to routine histopathological assessment. However, there is no established cutoff value that can reliably distinguish tumors with intermediate Ki67 labelling indices. In contrast, the strong and diffuse positive SP174 immunoreactivity of CAAs reported here offers a simple, easily interpreted binary distinction in which reactivity is either positive or negative, thus minimizing subjectivity, which is an inherent limitation with semi-quantitative scoring schemes commonly used for other IHC applications. Additionally, as shown in some of the cases in this report, CAA sections that contain non-neoplastic, normal surface gingival epithelium serve as an internal negative control minimizing interpretative bias.

Apart from CAA confirmation, the RAS Q61R-specific SP174 antibody may be used in the context of precision medicine, as proposed for human medical oncology (23–26). Arguably, rapid detection of mutated genes using IHC allows attending clinicians to rationally select targeted therapies for neoplasms known to harbor *HRAS*, *NRAS* or *KRAS* p.Q61R mutations including ameloblastoma, melanoma, colorectal, urothelial and thyroid cancers, among others (35). A highly specific and comparable diagnostic approach is available using the monoclonal antibody ‘VE1’ for detection of *BRAF* p.V600E in human cancer (23, 36, 37). Given that homologous encoding somatic mutations (i.e., *BRAF* p.V595E) are highly recurrent in some tumors of dogs, including papillary OSCC and urothelial carcinoma (20, 27, 38), VE1 IHC might be a useful tool that would complement RAS Q61R-specific SP174 antibody IHC. However, the use of VE1 for detection of mutated BRAF protein in dogs has yet to be validated. Indeed, our attempts to optimize VE1 IHC protocols using OSCC tumors confirmed to harbor a *BRAF* p.V595E mutation have been unsuccessful despite a high degree of sequence homology (data not shown). Although a cause for the lack of VE1 antibody reactivity with the corresponding mutated protein in canine oral tumors is uncertain, we speculate that level expression of the mutated *BRAF* allele may be below the detection limit of IHC, which could account for the lack of reactivity.

As reported herein, reliable detection of RAS Q61R with SP174 antibody in tumor tissues is possible using FFPE tissues that have not been decalcified together with optimization of IHC parameters, which may vary between various protocols, reagents, and laboratory equipment. Ideally, optimization of IHC protocols should be done using CAA tumors with a confirmed *HRAS* p.Q61R mutation as positive control, and normal oral or OSCC tumor tissues not harboring the mutation as negative controls. Additionally, because only FFPE tissues that were not decalcified were used in our study, the sensitivity and specificity of IHC staining with the RAS Q61R-specific SP174 antibody with tissues that may have been decalcified for various durations is unknown. Finally, even though recurrent *HRAS* p.Q61R mutations in oral tissues of dogs have only been demonstrated in CAA, they are not necessarily exclusive to this oral tumor type and thus additional comparative studies are required to determine the extent to which SP174 allows distinction between CAA and other proliferative lesions and normal oral epithelium.

In conclusion, this study demonstrates that IHC with RAS Q61R-specific SP174 antibody reagent is a highly sensitive, relatively simple, and specific method for confirmation of CAAs harboring an *HRAS* p.Q61R mutation.

## Data availability statement

The raw data supporting the conclusions of this article will be made available by the authors, without undue reservation.

## Ethics statement

The project referenced above has been approved by the Cornell University Veterinary Clinical Studies Committee (CUVCSC) following an ethical and scientific review. The project was exempted from review by the Cornell University Institutional Animal Care and Use Committee (IACUC) office because approval by the IACUC office was unnecessary for use of archival tissues. Owner consent for research use of such samples is signed by every client who consents to any treatment at the Cornell University Hospital for Animals.

## Author contributions

SP: Conceptualization, Data curation, Formal analysis, Funding acquisition, Investigation, Methodology, Project administration, Supervision, Visualization, Writing – original draft. MM: Investigation, Writing – review & editing. WK: Conceptualization, Funding acquisition, Resources, Writing – review & editing. GD: Conceptualization, Investigation, Methodology, Supervision, Validation, Writing – review & editing.
